# Association of COL1A1 rs1800012 polymorphism with musculoskeletal degenerative diseases: a meta-analysis

**DOI:** 10.18632/oncotarget.20797

**Published:** 2017-09-08

**Authors:** Binlong Zhong, Donghua Huang, Kaige Ma, Xiangyu Deng, Deyao Shi, Fashuai Wu, Zengwu Shao

**Affiliations:** ^1^ Department of Orthopaedic Surgery, Union Hospital, Tongji Medical College, Huazhong University of Science and Technology, Wuhan 430022, People’s Republic of China

**Keywords:** COL1A1 polymorphism, rs1800012, musculoskeletal degenerative diseases, osteoarthritis, intervertebral disc degeneration

## Abstract

It has been reported that the single nucleotide polymorphism (SNP) rs1800012 in COL1A1 gene might be linked to the susceptibility of musculoskeletal degenerative diseases, such as osteoarthritis (OA) and intervertebral disc degeneration (IVDD). However, the data from different studies is contradictory. Here we aimed to comprehensively summarize and clarify the relationship between the SNP and musculoskeletal degenerative diseases. Seven eligible studies including 1339 cases and 5406 controls were screened out from PubMed, Web Of Science and Cochrane library databases. Significant association was identified in sub group analysis of IVDD in homozygote model (GG versus TT: OR = 0.33, 95% CI 0.14–0.78, *P* = 0.012), heterozygote model (GT versus TT: OR = 0.29, 95% CI 0.11–0.72, *P* = 0.008) and dominant model (GG/GT versus TT: OR = 0.31, 95% CI 0.13–0.74, *P* = 0.008). Additionally, significant relationship was also found in sub group analysis of severe degree of IVDD in homozygote model (GG versus TT: OR = 0.37, 95% CI 0.15–0.91, *P* = 0.031), heterozygote model (GT versus TT: OR = 0.33, 95% CI 0.13–0.87,*P* = 0.024) and dominant model (GG/GT versus TT: OR = 0.36, 95% CI 0.14–0.88, *P* = 0.025). Although no significance was observed, there is a trend that the more G allele at COL1A1 rs1800012 site, the less possibility of IVDD and severe IVDD would happen. Our results indicate that COL1A1 rs1800012 polymorphism associates with the susceptibility of IVDD. However, this polymorphism may not be associated with OA risk.

## INTRODUCTION

Type I collagen, which is a heterotrimer constituted by 2α_1_ chains and 1α_2_ chain, is the major protein in skin, ligaments, and bone [1]. It can be roughly classified into type I collagen 1 (COL1A1) and type I collagen 2 (COL1A2). COL1A1 gene, located on chromosome 17 at position 17q21.33.7, encodes the α_1_ chain of type I collagen [2]. COL1A1 gene has arisen as a vital candidate gene of interest in many diseases recently. The relationship between gene polymorphism of COL1A1 gene and osteoporosis is mostly reported [3, 4]. Some articles also found the connection between COL1A1 gene and other diseases in musculoskeletal system, such as sports-related tendon and ligament injuries [5], osteogenesis imperfecta [6, 7], osteosarcoma [8], idiopathic carpal tunnel syndrome [9]. And among various polymorphisms within the COL1A1 gene, the most frequently studied polymorphism is the +1245G/T polymorphism (rs1800012, Sp1), which is a G to T polymorphism siting within the first intron of COL1A1 gene influencing a binding site for the transcription factor Sp1 [4, 10].

Intervertebral disc degeneration (IVDD) and osteoarthritis (OA) are common musculoskeletal disorders that cause pain, physical limitations and disability in later life. OA, a type of joint disease, is due to the destruction of joint cartilage and subchondral bone and is traditionally considered to be resulted from articular cartilage degeneration [11]. However, recent studies have demonstrated that the changes in the subchondral bone, whose extracellular matrix collagen is mainly composed by COL1A1, might play a pivotal role in the induction and progression of OA [12, 13]. Furthermore, it is also reported that genetic factors play an indispensable role in the development of OA [14, 15]. IVDD which results from ageing, natural daily pressure and minor injuries on intervertebral disc (IVD), has been considered as one of the primary causes to low back pain and motor deficiency. IVD is composed of three distinct parts: the central gelatinous nucleus pulposus, the outer annulus fibrosus (AF), and the cartilaginous endplate that anchors onto the vertebral body. Prevalence rate of IVDD and OA is rather high and increases with the age [16–20]. The most important things are that they are all degenerative diseases of musculoskeletal system partly resulting from aging or structural damage and that the extracellular matrix of subchondral bone and AF are mainly composed of COL1A1 [11, 21]. The mutations in COL1A1 gene may cause histology changes of subchondral bone in TMJ cartilage or knee joint and AF in IVD, leading to these two types of musculoskeletal degenerative diseases occurrences. So it makes us hypothesize that there may be a relationship between degenerative diseases of musculoskeletal system (OA and IVDD) and polymorphism of COL1A1 gene.

Actually, there do be several studies that have reported the association of musculoskeletal degenerative diseases with COL1A1 rs1800012 polymorphism. However, these studies have obtained conflicting results. Lian K et al. [22] and Luo S et al. [23] reported a positive relationship between incidence of OA or IVDD and G allele at rs1800012 site. Suprisingly, Tilkeridis et al. [24] and Pluijm et al. [25] got an opposite conclusion. Whereas the findings of Aerssens et al. [26]**,** Loughlin et al. [27] and Anjankar et al. [28] showed no significant association of OA or IVDD with rs1800012. However, there is no meta-analysis investigating the association between musculoskeletal degenerative diseases and COL1A1 rs1800012 polymorphism up to now. Therefore, we performed a meta-analysis to evaluate the association between them. In this study, we focus on identifying the connection of genetic mutations with musculoskeletal degenerative diseases, which seems to be of great importance and could help to forecast musculoskeletal degenerative risk for specific individuals or guide the clinical treatment of ‘high-risk’ individuals.

## RESULTS

### Characteristics of studies

**Figure 1:**
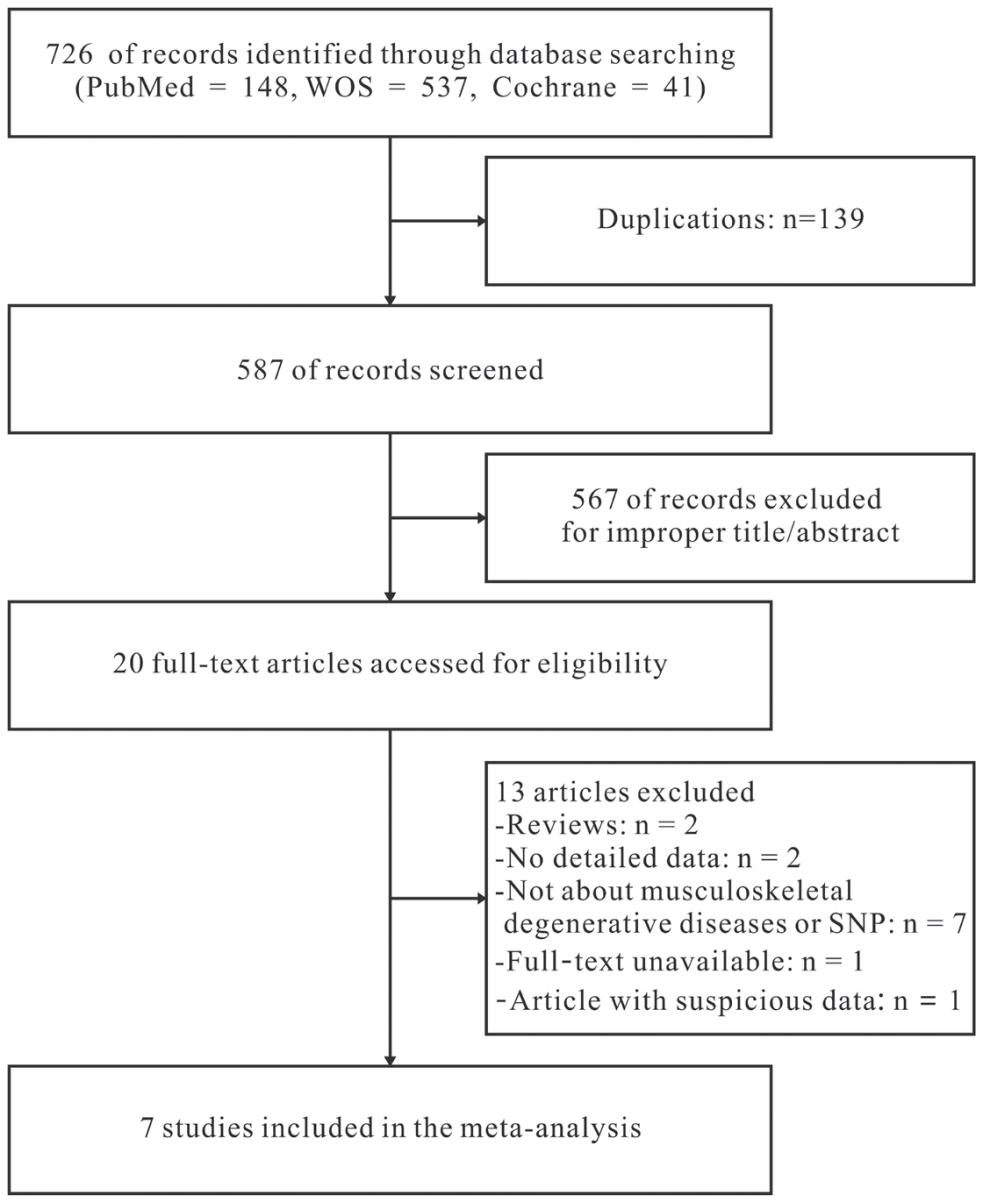
Flow diagram for the selection of studies

A total of 726 studies were acquired from PubMed, Web Of Science and Cochrane library databases (PubMed = 148, WOS = 537, Cochrane = 41). The literature selection process was shown in Figure 1. After removing the duplicates and browsing title and abstract, a total of twenty potential relevant studies were screened out. Then, further elimination of thirteen articles (two were reviews, seven focus on other diseases or polymorphism site, one cannot get the full article, two contained no detail data of genotype frequencies and one provided doubtful data) were performed through full text reading. No additional articles were selected through the manual search. Finally, seven studies [22–28] containing 1339 cases and 5406 controls fulfilled the predefined inclusion criteria and were included in the final analysis. The concrete characteristics of included studies were shown in Table 1. Among the seven studies, there were three IVDD studies, two osteoarthritis of the hip (OAH) studies, one temporomandibular joint osteoarthritis (TMJ OA) study and one hip or knee for OA. Although the percentage of COL1A1 in TMJ cartilage is markedly higher than hip or knee joint in anatomy and histology, we take them together when analyzing the relationship between COL1A1 rs1800012 polymorphism and OA since these three OA have similar pathogenesis in subchondral bone [12, 29–31]. All the IVDD patients were radiologically diagnosed and confirmed, and all the studies of OA other than Aerssens et al. [26], in which diagnosis method was not detailed, were radiologically diagnosed with or without clinical or histological examinations. All the participants in the control groups were healthy participants, functionally independent, not institutionalized and free from diseases or medications which might potentially affect the musculoskeletal system or the somatotropic axis. The genotyping distribution was claimed in agreement with Hardy-Weinberg equilibrium (HWE) in all studies.

**Table 1:** Characteristics of individual studies for associations between COL1A1 rs1800012 polymorphism and IVDD and OA risks

Study ID	Year	Country	Sex	Mean age (year)	Age range (year)	Diagnosis	Disease-level	Case			Control			P for HWE	Quality
								GG	GT	TT	GG	GT	TT		
Pluijm et al.	2004	Netherlands	both	75.6	65 or older	IVDD	severe	82	28	8	264	102	9	0.82	7
Tilkeridis et al.	2005	Greek	only describe young soldiers			IVDD	unknown	6	10	8	4	8	0	0.08	4
Anjankar et al.	2015	India	both	42.7	18–60	IVDD	severe	38	10	2	39	10	1	0.71	7
Aerssens et al.	1998	Belgium	female	73.8	60–90	OAH	severe	41	32	2	151	73	15	0.14	7
Lian K et al.	2005	US	female	78.5	65 or older	OAH	mild or moderate	158	74	14	2726	1291	158	0.74	7
							severe	224	97	4					
Luo S et al.	2016	China	both	35.2	16–79	TMJ OA	unknown	65	50	15	69	91	26	0.65	7
Loughlin et al.	2000	England	both	73.0	56–90	OAH and OAK	severe	233	120	18	243	116	10	0.38	6

### Association between COL1A1 rs1800012 polymorphism and musculoskeletal degenerative diseases

For all of the genetic models, random-effects model was used due to presence of heterogeneity. Although heterogeneity appeared in all models when we analyzed OA and IVDD together, analyzing OA or IVDD separately showed little heterogeneity in most of models. Overall, no significant association was identified in all models when analyzing all of the studies together (Table 2). For homozygote model (GG versus TT: OR = 0.84, 95% CI 0.47–1.48, *P* = 0.540). For dominant model (GG/GT versus TT: OR = 0.80, 95% CI 0.47–1.38, *P* = 0.427). For allele model (G versus T: OR = 0.97, 95% CI 0.81–1.16, *P* = 0.721). For heterozygote model (GT versus TT: OR = 0.77, 95% CI 0.44–1.34, *P* = 0.359). For recessive model (GG versus GT/TT: OR =1.01, 95% CI 0.84–1.22, *P* = 0.909).

**Table 2:** Meta-analysis results of the association between COL1A1 rs1800012 polymorphism and risk of IVDD and OA

COL1A1	*N*	G/T		GG vs TT		GT vs TT		GG/GT vs TT		GG vs GT/TT	
		OR	*P*	OR	*P*	OR	*P*	OR	*P*	OR	*P*
Overall	7	0.97 (0.81,1.16)	0.721	0.84 (0.47,1.48)	0.540	0.77 (0.44,1.34)	0.359	0.80 (0.47,1.38)	0.427	1.01 (0.84,1.22)	0.909
IVDD	3	0.78 (0.56,1.08)	0.130	0.33 (0.14,0.78)	0.012	0.29 (0.11,0.72)	0.008	0.31 (0.13,0.74)	0.008	0.92 (0.62,1.37)	0.690
OA	4	1.03 (0.85,1.26)	0.752	1.14 (0.68,1.88)	0.623	1.02 (0.63,1.64)	0.939	1.07 (0.68,1.69)	0.758	1.03 (0.79,1.36)	0.813

**Figure 2:**
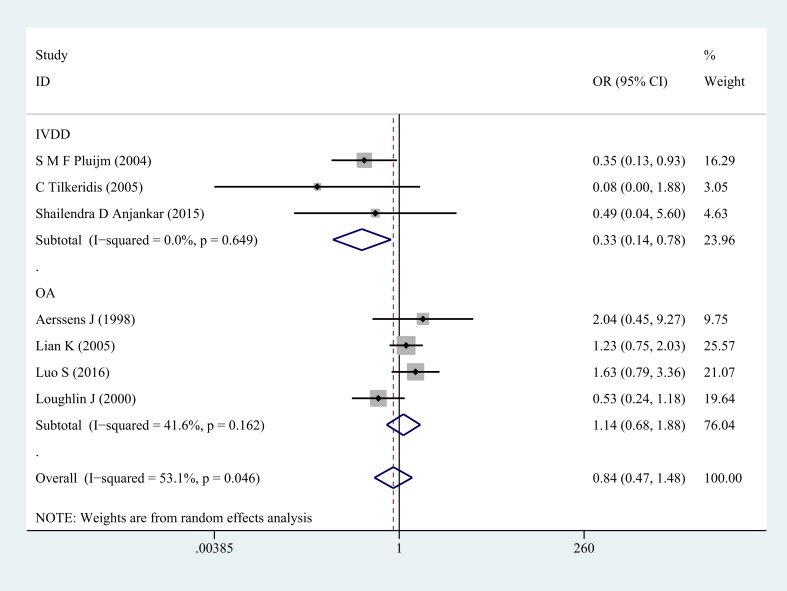
Forest plot of homozygote comparison of COL1A1 rs1800012 in musculoskeletal degenerative diseases for subgroup analysis stratified by diagnosis (GG versus TT)

**Figure 3:**
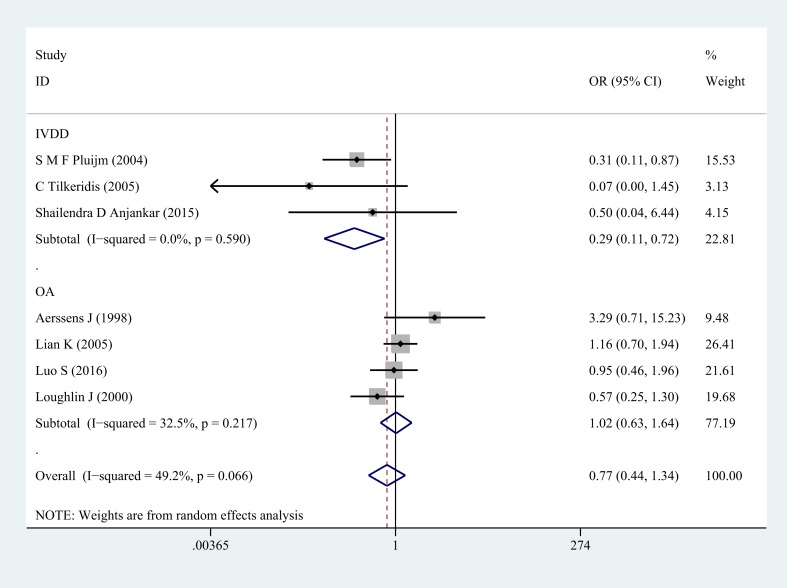
Forest plot of heterozygote comparison of COL1A1 rs1800012 musculoskeletal degenerative diseases for subgroup analysis stratified by diagnosis (GT versus TT)

**Figure 4:**
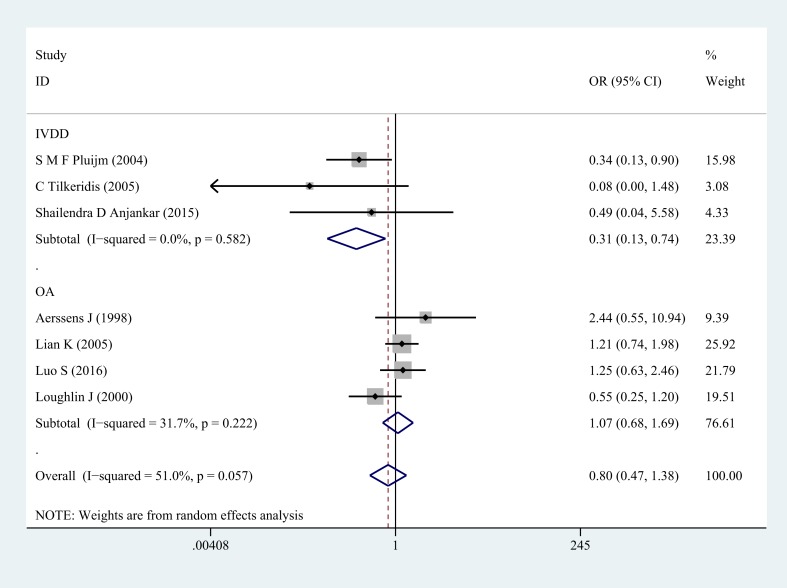
Forest plot of dominant comparison of COL1A1 rs1800012 musculoskeletal degenerative diseases for subgroup analysis stratified by diagnosis (GG/GT versus TT)

Next, subgroup analysis was implemented according to different diagnoses. The association of COL1A1 rs1800012 polymorphism with the risk of musculoskeletal degenerative diseases was analyzed in two independent studies. In IVDD, significant association was found in homozygote model (GG versus TT: OR = 0.33, 95% CI 0.14–0.78, *P* = 0.012 Figure 2), heterozygote model (GT versus TT: OR = 0.29, 95% CI 0.11–0.72, *P* = 0.008 Figure 3) and dominant model (GG/GT versus TT: OR = 0.31, 95% CI 0.13–0.74, *P* = 0.008 Figure 4), but no significant association was found in recessive model (GG versus GT/TT: OR = 0.92, 95% CI 0.62–1.37, *P* = 0.690) or in allele model (G versus T: OR = 0.78, 95% CI 0.56–1.08, *P* = 0.130). In OA, no significant association was found in homozygote model (GG versus TT: OR = 1.14, 95% CI 0.68–1.88, *P* = 0.623), heterozygote model (GT versus TT: OR = 1.02, 95% CI 0.63–1.64, *P* = 0.939), dominant model (GG/GT versus TT: OR = 1.07, 95% CI 0.68–1.69, *P* = 0.758), recessive model (GG versus GT/TT: OR =1.03, 95% CI 0.79–1.36, *P* = 0.813) or allele model (G versus T: OR = 1.03, 95% CI 0.85–1.26, *P* = 0.752).

### Association between COL1A1 rs1800012 polymorphism and severe musculoskeletal degenerative diseases

Some of the articles included in the present study describe the level of diseases in their case group. We defined the severe degree of diseases as the patient who had a Kellgren score of 3 or greater in IVDD [28, 32] and the patient who had a joint space narrowing(JSN) score ≥ 3 in OA [33, 34] or took a joint replacement due to OA. We defined the mild or moderate degree of diseases as the patient who suffered OA or IVDD but did not satisfy the criterion mentioned above. Because of the limited data of mild or moderate degree, we only analyzed the relationship between COL1A1 rs1800012 polymorphism and severe degree of musculoskeletal degenerative diseases. Five articles [22, 25–28] were included.

For recessive model, fixed-effects model was used, because no significant heterogeneity was identified by I-squared statistic. For other models, random-effects model was used due to presence of heterogeneity. Overall, no significant association was identified in all of models when analyzing all of the studies together (Table 3). For homozygote model (GG versus TT: OR = 0.92, 95% CI 0.35–2.44, *P* = 0.867). For dominant model (GG/GT versus TT: OR = 0.94, 95% CI 0.35–2.54, *P* = 0.909). For heterozygote model (GT versus TT: OR = 0.98, 95% CI 0.35–2.74, *P* = 0.976). For recessive model (GG versus GT/TT: OR =0.99, 95% CI 0.85–1.17, *P* = 0.942). For allele model (G versus T: OR = 0.96, 95% CI 0.78–1.17, *P* = 0.685).

**Table 3:** Meta-analysis results of the association between COL1A1 rs1800012 polymorphism and risk of severe IVDD and OA

COL1A1	*N*	G/T		GG vs TT		GT vs TT		GG/GT vs TT		GG vs GT/TT	
		OR	*P*	OR	*P*	OR	*P*	OR	*P*	OR	*P*
Overall	5	0.96 (0.78,1.17)	0.685	0.92 (0.35,2.44)	0.867	0.98 (0.35,2.74)	0.976	0.94 (0.35,2.54)	0.909	0.99 (0.85,1.17)	0.942
IVDD	2	0.83 (0.59,1.18)	0.299	0.37 (0.15,0.91)	0.031	0.33 (0.13,0.87)	0.024	0.36 (0.14,0.88)	0.025	0.95 (0.63,1.42)	0.785
OA	3	0.99 (0.75,1.31)	0.963	1.14 (0.40,5.18)	0.575	1.63 (0.47,5.73)	0.443	1.52 (0.43,5.40)	0.522	1.00 (0.84,1.20)	0.968

**Figure 5:**
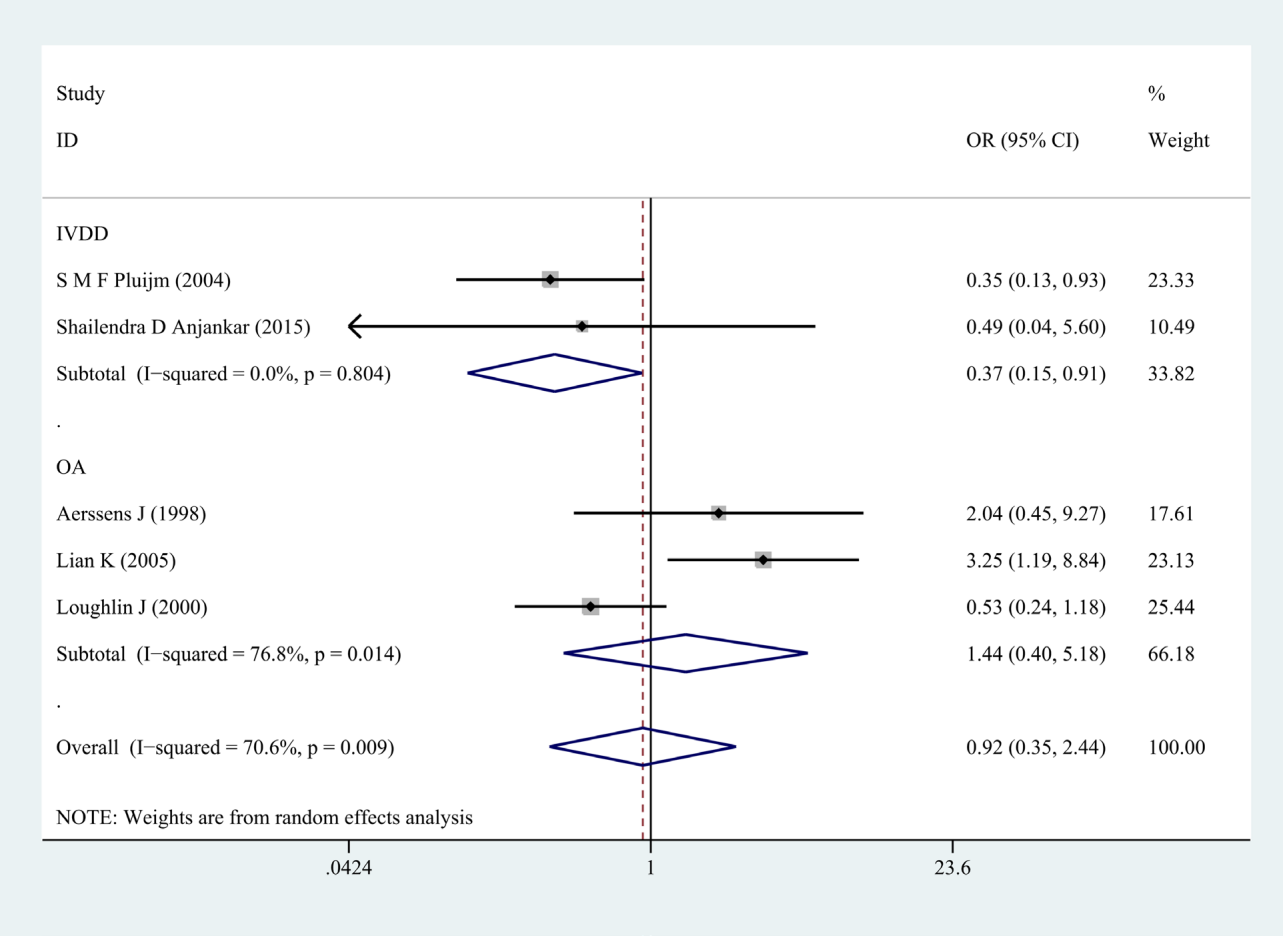
Forest plot of homozygote comparison of COL1A1 rs1800012 severe musculoskeletal degenerative diseases for subgroup analysis stratified by diagnosis (GG versus TT)

**Figure 6:**
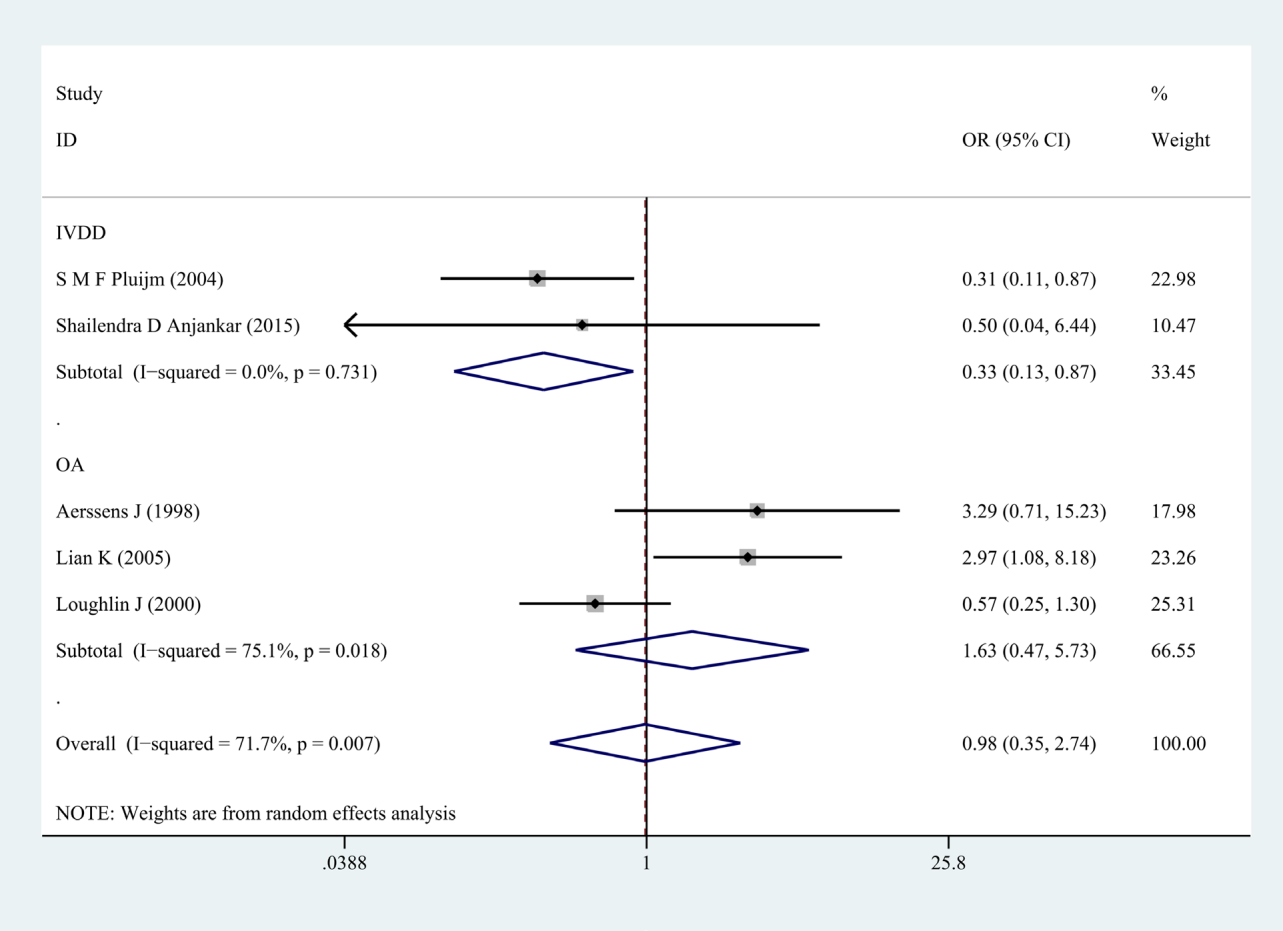
Forest plot of heterozygote comparison of COL1A1 rs1800012 severe musculoskeletal degenerative diseases for subgroup analysis stratified by diagnosis (GT versus TT)

**Figure 7:**
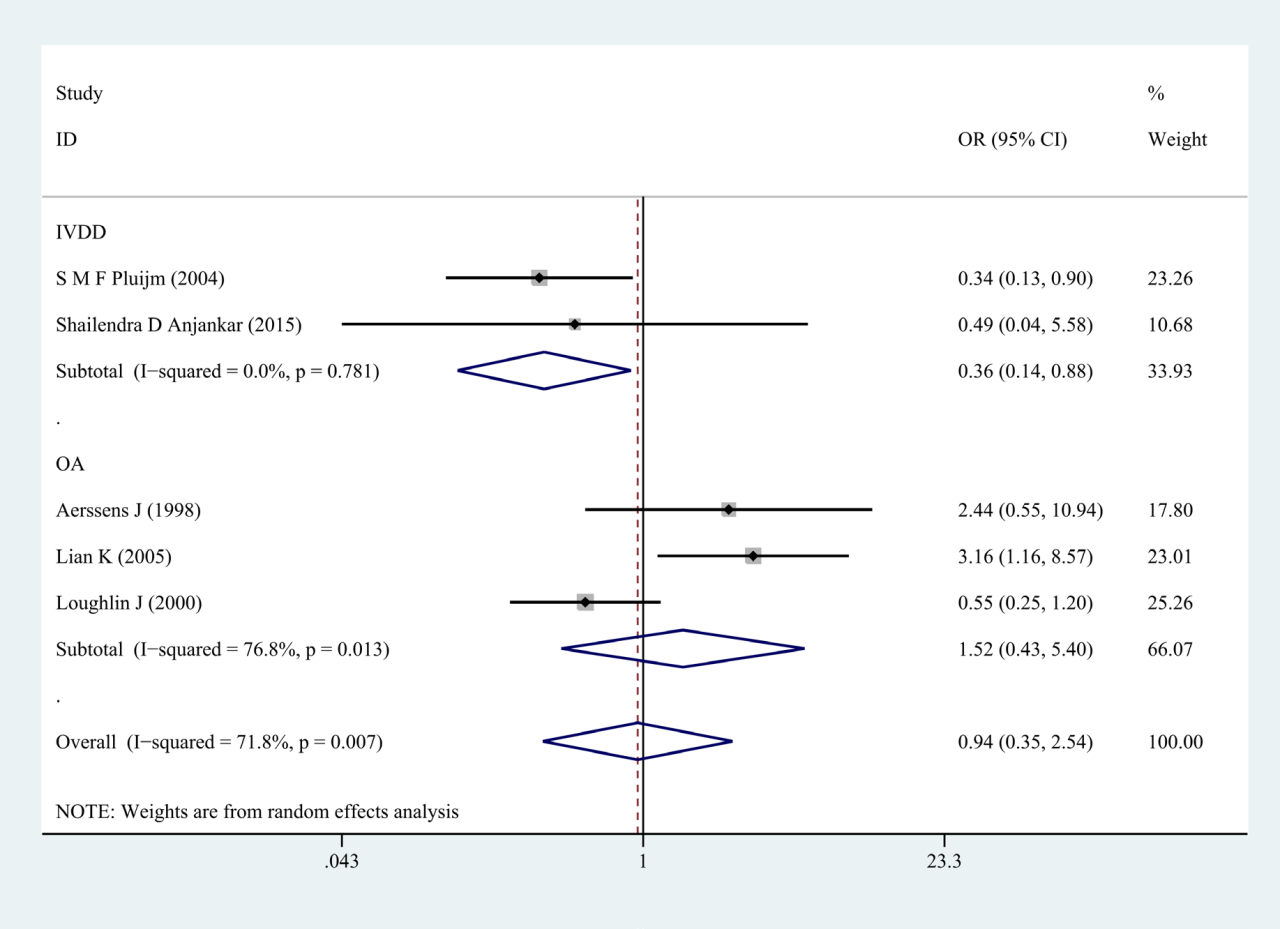
Forest plot of dominant comparison of COL1A1 rs1800012 severe musculoskeletal degenerative diseases for subgroup analysis stratified by diagnosis (GG/GT versus TT)

Next, subgroup analysis was conducted according to different diagnoses. The association between COL1A1 rs1800012 polymorphism and the risk of severe musculoskeletal degenerative diseases was analyzed in two independent studies. In IVDD, significant association was found in homozygote model (GG versus TT: OR = 0.37, 95% CI 0.15–0.91, *P* = 0.031 Figure 5), heterozygote model (GT versus TT: OR = 0.33, 95% CI 0.13–0.87, *P* = 0.024 Figure 6) and dominant model (GG/GT versus TT: OR = 0.36, 95% CI 0.14–0.88, *P* = 0.025 Figure 7), but no significant association was found in allele model (G versus T: OR = 0.83, 95% CI 0.59–1.18, *P* = 0.299) or in recessive model (GG versus GT/TT: OR =0.95, 95% CI 0.63–1.42, *P* = 0.785). In OA, no significant association was found in homozygote model (GG versus TT: OR = 1.14, 95% CI 0.40–5.18, *P* = 0.575), heterozygote model (GT versus TT: OR = 1.63, 95% CI 0.47–5.73, *P* = 0.443), dominant model (GG/GT versus TT: OR = 1.52, 95% CI 0.43–5.40, *P* = 0.522), recessive model (GG versus GT/TT: OR =1.00, 95% CI 0.84–1.20, *P* = 0.968) or allele model (G versus T: OR = 0.99, 95% CI 0.75–1.31, *P* = 0.963).

### Sensitivity analysis and publication bias

Sensitivity analysis was performed to examine the effects set by individual study on the pooled ORs for COL1A1 rs1800012 polymorphism by deleting one study each turn in every genetic model [16]. No change in the significance of outcomes happened in all of the models. Because of the limited number (below 10) of studies included in each analysis, publication bias was not assessed.

## DISCUSSION

Musculoskeletal degenerative diseases, which include osteoporosis, IVDD and OA, are widely considered as multifactorial diseases causing a huge medical and economic burden to society. The association between COL1A1 rs1800012 polymorphism and the risk of osteoporosis as well as osteoporosis-induced fracture has been well established [3, 35]. In this study, we performed a meta-analysis to determine the relationship between COL1A1 rs1800012 polymorphism and the risk of IVDD and OA. The present meta-analysis included 7 case-control studies which enrolled a total of 1339 cases (192 IVDD patients and 1147 OA patients) and 5406 healthy controls. The merged ORs and 95% CI were carefully evaluated in five genetic models. It is interesting that we found no significant association between COL1A1 rs1800012 polymorphism and the risk of musculoskeletal degenerative diseases. In sub group analysis, we demonstrated significant associations of COL1A1 rs1800012 polymorphism with risk of IVDD and severe IVDD sub groups in homozygote model, heterozygote model and dominant model, in which the 95% CI did not overlap the lines of the pooling results. Although not significant, trend of protective effect of G allele was observed in allele model of IVDD and severe IVDD patients. However, no significant associations with COL1A1 rs1800012 polymorphism were noted in OA and severe OA subgroups.

The type I collagen is partly encoded by COL1A1 gene. The rs1800012 polymorphism located in the first intron of the COL1A1 gene has been identified as an important SNP in regulation of collagen transcription [10]. COL1A1 gene with G/T polymorphism affects the DNA-protein interaction and increases the levels α1 chains of type I collagen, resulting in an imbalance in the normal ratio of α1 to α2 chains (2:1), by heightening the binding affinity of RNA polymerase II [36, 37]. Literatures have demonstrated that COL1A1 rs1800012 polymorphism contribute to the risk of musculoskeletal diseases. A latest meta-analysis contained 1557 subjects has indicated that GG genotype in COL1A1 rs1800012 polymorphism shows a protective effect to osteoporosis risk in post-menopausal women [3]. Additionally, the COL1A1 rs1800012 polymorphism has been reported to be related to osteoporotic fractures by bone mass reduction [10]. For musculoskeletal diseases caused by connective tissue and soft tissue, like sports-related tendon and ligament injuries, TT genotype plays a protective role in the risk of these disorders, especially in anterior cruciate ligament injuries [5].

In this study, we evaluated the association between two common disorders of musculoskeletal degenerative diseases. IVDD is now considered as multifactorial degenerative disease caused by mechanical overloading, senescence, and environmental factors. The genetic factors has been confirmed to be associated with the susceptibility of IVDD, such as gene polymorphisms of aggrecan, insulin-like growth factor-1 receptor, interleukin 1, vitamin D receptor and matrix metalloproteinase 3 [38]. In this meta-analysis, we demonstrated that the COL1A1 rs1800012 polymorphism was associated with the risk of IVDD and severe IVDD strongly. Despite the significant association between COL1A1 rs1800012 polymorphism and the susceptibility of IVDD, no sufficient evidences in OA were observed. In the pathophysiology of osteoarthritis, degeneration of articulation was believed to be associated with breakdown of collagen fibers and changes in subchondral bone, which includes bone turnover enhancement, bone mass loss and sclerosis.[11, 12, 31] It should be noted that the major collagen of articular cartilage and subchondral bone are type II collagen and type I collagen, respectively. The association between polymorphism of COL2A1 gene and OA has been demonstrated in Chinese, Mexican and Finnish population [39–41]. Combining with the knowledge of the composition in articular cartilage and subchondral bone, we speculate the COL1A1 rs1800012 polymorphism and the change of subchondral bone may act as minor factors in genetic etiology of OA. In other words, the alternations of articular cartilage may still contribute a major role in OA development, as the previous articles mentioned. More studies need to be included to clarify this point in the future.

Our meta-analysis has several strengths. Firstly, to our knowledge, this is the first meta-analysis focused on the association between COL1A1 gene polymorphism and the susceptibility to musculoskeletal degenerative diseases, including OA and IVDD. And we also took the level of diseases into account. Moreover, multiple strategies and strict criteria were applied to assess the methodological quality of the studies; most of the recruited studies possessed high qualities and no study was calculated significantly deviated from Hardy-Weinberg equilibrium (HWE). The last but not the least, this study included researches of Asia (China and India), Europe (Netherlands, Greek, Belgium and England) and America (USA), containing different kinds of ethnicities. So the result is much more comprehensive.

The present meta-analysis also has a few limitations that should be taken into account. Firstly, the number of included studies for COL1A1 rs1800012 polymorphism limited further analysis due to shortage of original studies. Secondly, the heterogeneity was a little bit high when OA and IVDD were analyzed together, leading to a cautious acceptance of the results. So we analyzed OA and IVDD separately in subgroup to make the result more credible. Thirdly, some studies were excluded from our research because of lacking original data or having suspicious data, which may contribute to selection bias. What’s more, the included studies were observational studies and our results were based on unadjusted estimates. The confounding factors such as age, occupation and ethnicity may affect the results.

In conclusion, our results suggest that COL1A1 rs1800012 polymorphism associates with the susceptibility of IVDD. In contrast, this polymorphism may not be associated with OA risk. Considering the limitation of this meta-analysis, larger-scale studies, which include more populations and take confounding factors into account, are necessary to further explore the roles of COL1A1 gene in the pathogenesis of musculoskeletal degenerative diseases.

## MATERIALS AND METHODS

### Identification of eligible studies

Relevant available literature published in PubMed, Web Of Science and Cochrane library databases was searched by two investigators independently. The final literature search was performed on Jun 15, 2017. The following terms were used: (“COL1A1” or “type I collagen alpha 1” or “procollagen alpha1(I)” or or “sp1” or “rs1800012”) and (“Intervertebral Disk Degeneration” or “Disk Degeneration, Intervertebral” or “IDD” or “Disc Degeneration” or “disc herniation” or “low back pain”) and (“Osteoarthritis” or “Osteoarthritides” or “Osteoarthrosis” or “Arthritis, Degenerative” or “Degenerative Arthritides” or “Degenerative Arthritis” or “Osteoarthrosis Deformans”). To avoid incomplete retrieving, the bibliography of recent related reviews and the primary articles were manually searched for all identified studies.

### Inclusion and exclusion strategy

The following inclusion strategies were used: (1) Evaluation of the association between COL1A1 Sp1 gene polymorphism and the risk of IVDD or OA; (2) Human subjects; (3) Case-control study; and (4) Available genotype frequencies data was provided so that the odds ratios (ORs) and 95% confidence intervals (CIs) could be calculated.

Correspondingly, the exclusion criteria were defined as: (1) Reviews, comments or animal studies; (2) Data overlapping with previous publications; (3) Family-based design studies; (4) Studies without available genotype frequencies and cannot be obtained by contact with authors.

All retrieved literatures were assessed and discussed to achieve consensus by two investigators according to the inclusion and exclusion strategy independently.

### Data extraction

The following information was carefully collected by two investigators from all qualified studies independently: (1) Name of the first author; (2) Year of publication; (3) Nation where the study was conducted; (4) Gender and age of enrolled subjects; (5) Numbers of case group and control group; (6) Genotyping method; (7) Genotype frequency in case group and control group; and (8) Diagnosis and the disease-level. The two authors reached a consensus on all the data.

### Methodological quality assessment

The two investigators evaluated the qualities of the included studies independently by using the Clark scores system, which contains 10 items [42]. Scores below 5 indicate low quality, while 5–7 scores denote moderate quality and 8–10 scores represent high quality [42].

### Statistical analysis

The PRISMA checklists and their guidelines were carefully followed in the whole process of this study.[43] The association strength between COL1A1 Sp1 polymorphism and IVDD or OA risk was evaluated by merging ORs with 95% CI. The estimations of pooled ORs were determined by the weighted average OR from each study. A significant risk was determined by a *P-value* less than 0.05 in *Z*-test. The pooled ORs were calculated in homozygote model (GG versus TT), heterozygote model (GT versus TT), dominant model (GG/GT versus TT), and recessive (GG versus TT/GT) model respectively. To determine statistical heterogeneity, Q statistic and I^2^ statistic was evaluated in each model. Significant heterogeneity was considered when *P* < 0.10 and *I*^2^ > 50% [44]. A random-effects model was used if significant heterogeneity existed; in other case, the fixed-effects model was chosen [45]. Sensitivity analyses were performed to evaluate the effect of an individual study on the combined ORs by removing each study in turn. All analyses were performed using STATA 14 (Stata, CollegeStation, TX). All *P*-values were two-sided.

## Abbreviations

AF: annulus fibrosus
CIs: confidence intervals
COL1A1: type I collagen alpha 1
COL1A2: type I collagen alpha 2
HWE: Hardy-Weinberg equilibrium
IVD: Intervertebral disc
IVDD: intervertebral disc degeneration
LSN: joint space narrowing
OA: osteoarthritis
OAH: osteoarthritis of the hip
OAK: osteoarthritis of the knee
ORs: odds ratios
SNP: single nucleotide polymorphism
TMJ OA: temporomandibular joint osteoarthritis
